# Bone Loss at Implant with Titanium Abutments Coated by Soda Lime Glass Containing Silver Nanoparticles: A Histological Study in the Dog

**DOI:** 10.1371/journal.pone.0086926

**Published:** 2014-01-22

**Authors:** Arturo Martinez, Francisco Guitián, Roberto López-Píriz, José F. Bartolomé, Belén Cabal, Leticia Esteban-Tejeda, Ramón Torrecillas, José S. Moya

**Affiliations:** 1 Facultad Medicina y Odontología, Universidad de Santiago de Compostela (USC), Galicia, Spain; 2 Instituto de Cerámica de Galicia, Universidad de Santiago de Compostela (USC), Galicia, Spain; 3 Nanomaterials and Nanotechnology Research Center (CINN-CSIC) – Universidad de Oviedo (UO) – Principado de Asturias, Llanera, Spain; 4 Instituto de Ciencia de Materiales de Madrid (ICMM-CSIC), Madrid, Spain; University of Toronto, Canada

## Abstract

The aim of the present study was to evaluate bone loss at implants connected to abutments coated with a soda-lime glass containing silver nanoparticles, subjected to experimental peri-implantitis. Also the aging and erosion of the coating in mouth was studied. Five beagle dogs were used in the experiments. Three implants were placed in each mandible quadrant: in 2 of them, Glass/n-Ag coated abutments were connected to implant platform, 1 was covered with a Ti-mechanized abutment. Experimental peri-implantitis was induced in all implants after the submarginal placement of cotton ligatures, and three months after animals were euthanatized. Thickness and morphology of coating was studied in abutment cross-sections by SEM. Histology and histo-morphometric studies were carried on in undecalfied ground slides. After the induced peri-implantitis: 1.The abutment coating shown losing of thickness and cracking. 2. The histometry showed a significant less bone loss in the implants with glass/n-Ag coated abutments. A more symmetric cone of bone resorption was observed in the coated group. There were no significant differences in the peri-implantitis histological characteristics between both groups of implants. Within the limits of this in-vivo study, it could be affirmed that abutments coated with biocide soda-lime-glass-silver nanoparticles can reduce bone loss in experimental peri-implantitis. This achievement makes this coating a suggestive material to control peri-implantitis development and progression.

## Introduction

Peri-implantitis has been cited as one of the key factors responsible for implant failure [1], [2]. It is defined as an infectious disease characterized by crestal bone (CrB) loss and bleeding on probing with or without deepening on peri-implant pockets [3]. Nowadays it is well accepted that peri-implantitis is a process that involves microorganisms similar to those found in chronic peridontitis as found around teeth [4].

Some strategies have been developed in the peri-implantitis treatment in recent years [5]: i) prevention of bone loss around implants. In this regards new implant designs have been commercialized seeking to reduce bone remodeling after osseointegration as well as modern implant abutment connection (eg. morse cone-connection) minimizing bacterial filtration- although due to the impossibility of completely eliminating bacterial contamination, subgingival plaque formation is still a problem which often result in peri-implantitis; and ii) treatment based on mechanical debridation, antibiotic treatment and osseous regeneration when possible [6]. The use of local antibiotics and antiplaque biocides, in addition to manual debridement seems to be an adequate treatment [7], [8]. However, it seems that the eradication of resistance is impossible and development of resistance to any particular antibiotic is inevitable.

A new approach to biomedical device-associated infections is based on biocide materials [9]. Silver as a nonspecific biocide agent is able to act strongly against a broad spectrum of bacterial and fungal species, including antibiotic-resistant strains. It is believed that silver nanoparticles (Ag NPs) are more reactive than bulk metallic forms because of the more active sites that result from a high specific surface [10], [11]. There is evidence that the sealing of soft tissue on the implant surface plays a role in the prevention of peri-implantitis [12], [13], [14]. While it is true no unanimity exists about this point in the literature. It is believed that the transmucosal elements should have a polished surface to prevent adhesion of biofilm [15], [16], [17]. In this regard the use of a coating that can reduce bacterial activity in peri-implant tissue is an interesting topic, since lead to a greater stability of the gingival seal.

In this investigation, we have tested a sodalime-glass containing Ag NPs-coated titanium healing abutments in an experimental peri-implantitis model. The experimental peri-implantitis, described in the literature, reproduces an infectious process leading to bone loss [18], [19]. The present work focuses on two hypotheses: The null hypothesis (1) is that the transmucosal abutment biocide coating, under experimental peri-implantitis, do not experience dimensional changes while in mouth. The null hypothesis (2) is that the use of the biocidal coating on the surface of the transepithelial abutments does not reduce bone loss or alter tissue response versus experimental peri-implantitis.

## Materials and Methods

### Material

We have used a Soda-Lime-Glass/nAg powder to perform the coating on Ti-6Al-4V alloy. The preparation of the starting powder and the characterization of the coatings were carried out according to the method developed by Esteban-Tejeda et al [10]. Homogeneous dispersed silver nanoparticles embedded into glassy matrix, with a content of silver of 20 wt.%, have been obtained as described below: A commercial soda-lime glass with the following chemical composition (mol.%): 70.30 SiO_2_, 0.92 B_2_O_3_, 15.34 Na_2_O, 7.62 CaO, 0.03 K_2_O, 4.78 MgO, 1.01 Al_2_O_3_, 0.01 Fe_2_O_3_, and the corresponding fraction of vitellinate-nAg [i.e., commercial protein with silver nanoparticles (batch n° 127, ARGENOL S.L.)] were homogeneously blended in isopropyl alcohol overnight under constant stirring. After the suspensions were dried at 60°C for 4 h, the homogeneous mixtures were uniaxially pressed into pellets (Ø∼10 mm) at 250 MPa. Next, they were sintered in two steps by heating to 500°C and to 725°C (rate of 3°C/min and dwell of 1 h), in order to ensure a complete elimination of the organic compounds from the vitellinate. The obtained glass pellets were milled down to <32 µm in an agate planetary mortar. These obtained powders were characterized by XRD, UV-VIS spectroscopy, scanning electron microscopy (SEM) and transmission electron microscopy (TEM) [20].

The green coating was obtained by dipping the Ti6Al4V abutments (Phibo ProUnic model, Spain) into a pentanol (Fluka-1-pentanol, 98.0% purity) glass-nAg powder suspension with 70 wt. % solid content. Before dipping, the suspension was dispersed in an ultrasonic bath and with a magnetic stirrer. During the coating process, the abutments were vertically dipped into the suspension at a constant speed of 500 mm/min, immersed into the suspension for 3 seconds, and then withdrawn at the same speed. The resulting coatings were dried at room temperature (20°C) for 24 h. The green coated abutments were subsequently heated in an argon atmosphere at 980°C for 1 h.

Surface rugosity (Ra values) of uncoated abutments was estimated in 0.5±0.3 µm and 1±0.2 µm in the coated [21].

### Animal Study Design

**Figure 1:**
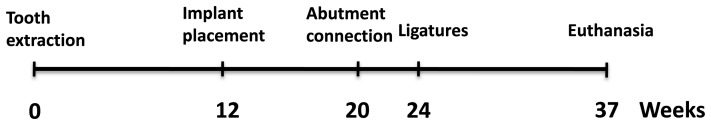
Outline of the study. After a period of two months healing abutments were placed. Ligatures were placed 4 weeks after. After a period of 3 months of active peri-implantitis the necropsy was done.

The dogs were purchased from Minimally Invasive Surgery Centre, Cáceres, Spain. The study protocol was approved by their Ethics Committee for Animal Research Welfare. Five 1 year old Beagle dogs (weight ranging from 12–15 Kg) were used. The outline of the experiment is presented in Fig. 1. During all procedures veterinary assistance was mandatory. General anesthesia was induced with intravenous injected propofol 10 mg/kg (Propofol Hospira, Hospira Productos Farmacéuticos y Hospitalarios, Madrid, Spain). A n°7 endotracheal tube with a balloon cuff was placed and connected to a circular anesthesia circuit (Leon Plus, Heinen & Löwenstein, Bad Ems, Germany). The anesthesia was sustained with sevofluorane (Sevorane, Abbott Laboratories, Madrid, Spain). Multimodal analgesia was employed in the perioperatory (ketorolac 1 mg/kg (Toradol 30 mg, Roche); - tramadol 1.7 mg/kg (Adolonta inyec., Grünenthal); y - buprenorfine 0,01 mg/kg (Buprex, Reckitt Benckiser Pharmaceuticals Limited, Berkshire, UK).

### Surgery

All mandibular premolars and the first molar were extracted. After three months of healing the possible difference in width (periosteal level) between mesial and distal, in the gap of the edentulous alveolar ridge, was determined. In this regard a spreading caliper (ACE Brock Mass REF. 080,052) was used to measure at periosteal level, the edentulous ridge thickness on both sides, one mesial and one as far distal (5 mm apart from gingival margin of the adjacent teeth) in the five studied animals. After conducting a paired t test significant difference between the measures with respect to right and left and no significant difference in measures between mesial and distal width were found (Table 1).

**Table 1:** Edentulous alveolar ridge width.

N	Left	Right	SIGNIFIC.
20	5.02±0.25	4.75±0.23	0.001
N	distal	mesial	
20	4.94±0.32	4.84±0.21	0.260

Mucoperiosteal flaps were raised and 3 fixtures (Phibo Dental Solutions®, Barcelona, Spain; TSA Advance: length 11.5, diameter 3.75) were installed in the edentulous region on both sides of the mandible. The occlusal surface of the implant was placed flush with the bone. A total of 30 implants were placed in the five dogs. During this period animals were feed with a soft diet. Two months later, abutment connection (ProUnic® Advance, Phibo; height 2 mm) was performed. As one of the objectives was to study the possible wear of the coating of the healing abutment, and bearing in mind the possible functional asymmetry [22] of the tongue and chewing [23], [24] and the width asymmetry detected (Table 1), the split-mouth design was not used. Given the absence of significant differences in peak width between the mesial and distal a fixed position for the abutments was chosen [25]. The mesial implants of each quadrant (position 4) supported a machined titanium healing abutment, and were considered controls (group A). The central (position 5) and distal (position 6) implants in each quadrant dressed biocide coated titanium healing abutments, and were considered case implants (group B1 and B2).

A plaque control program was initiated. This included cleaning of teeth and implants, once a day, 5 days a week, with toothbrush and dentifrice. The plaque control regimen was terminated four weeks later. At the end of the plaque control period, the animals were examined, and as it was expected [12], each group experimented a bone recession related to the biological width setting [21].

### Experimental Peri-implantitis

Four weeks after abutment connection, cotton ligatures were placed in a submarginal position around the neck of the fixture abutments according to the technique described by Ericsson et al [26] and Lindhe et al [27]. The plaque control regimen was finished and thus the plaque was allowed to accumulate during the course of the following three months. Once a week a clinical examination was performed to assess: the plaque, soft tissue inflammation and presence of ligature. The ligatures were substituted every three weeks with new ligatures placed in the pocket of the receded gingival margin. One implant from the B group was lost, due to a rapid progression of bone loss.

### Histological Preparation and Analysis

Animals were euthanized with a lethal dose of Sodium-Penthotal®, mandibular blocks containing fixtures were retrieved and stored in a 5% formaldehyde solution (pH 7). The implant blocks were retrieved from the jaw bone using an oscillating autopsy saw (Exakt, Kulzer, Germany). The dissected specimens were immediately immersed in a solution of 4% formaldehyde and 1% calcium and processed for ground sectioning following the Donath & Breuner methods [28]. Each implant block was individualized, embedded in methyl-methacrylate and stained with combined Harris Haematoxyline and Wheatley. Two central bucco-lingual ground slides of about 25 µm were obtained from each implant. The histological analysis was performed by using a transmitted light microscope (Optiphot 2-POL, Nikon, Japan) equipped with a digital camera (DP-12, Olympus, Japan).

### Coating Stability

The remaining, mesial and distal, resin block portions of 4 implants with coated abutments were polished (polishing diamond<1 µm) and were studied by RLOM and SEM (JEOL 6700, Japan), 20 measurements of coating thickness were performed in buccal and lingual sides. As a control group 20 thickness measurements were done in 3 unemployed coated abutments that were also resin embedded and polished. Means were compared with a t test.

### Histomorphometric Evaluation

**Figure 2:**
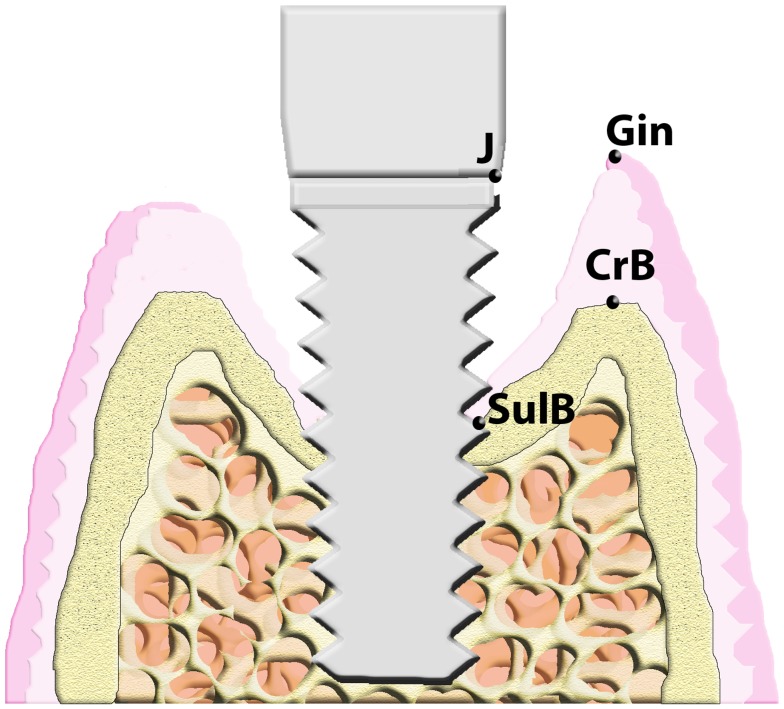
Landmarks employed in the histometric study. (J: abutment-fixture junction; SulB: coronal position of bone to implant contact; CrB: most coronal position of crestal bone; Gin: most coronal position of gingival margin).

Preparations of undecalcified thin ground slides were observed at 13x on an Olympus microscope SZX12 (Japan) and have been photographed using a special camera (DP-12, Olympus, Japan). On the images obtained following landmarks were identified and used in the measurements. The most occlusal point of the gingival margin (Gin), the abutment-fixture junction (J), the most occlusal point of the crestal bone (CrB) and the marginal possition of bone-implant contact (SulB). These measurements were performed on both buccal and lingual sides. Landmarks are shown in Fig. 2.

Mean values for each linear measurements were obtained for each implant. As independent variables were considered: i) the presence or absence of coating (A, B), ii) the position in the jaw (mesial, medial, distal), iii) the animal (1–5), iv) the side of the jaw (L, R) and v) if the measurement corresponds to buccal (B) or lingual (L) sides. Normality of the data according to each of the classification variables, were tested employing a Kolmogorof-Smirnov test (KS). The variables in which there were more than two groups were studied with a repeated measurements one-way ANOVA and then a post-hoc comparison between the three implant positions, with a paired t test, in each hemi-mandible, in order to define the significance between test (B1, B2) and the control (A) as well as the test itself. The null hypothesis was rejected at p≤0.05.

Slides were examined on an Olympus microscope. In order to quantify the degree of peri-implant lesion, presence or absence of five pathological aspects were established as categorical scores [4]: i) ulcerated pocket epithelium (UPE), ii) mucosal infiltration by inflammatory cells, iii) disruption of the collagen network (Coll) of submucosal, iv) intrabone pocket and v) vascular proliferation. Also in each of the preparations the proportion of lymphocytes, polymorphonuclear (PMN) and plasmocytes in a counting 100 cells in a field 100×100 µm from a typical subepithelial region were quantified. The difference in structure and appearance of peri-implant tissues was studied using a χ2 for the categorical scores and a one way ANOVA test was employed with the frequencies of various inflammatory cells. The level of significance was set at p≤0.05.

To assess the measurement error in all morphometric analysis (ME) two operators, independently, and with a 1 week of interval, performed two sets of replicated measurements, randomly and without information on the identification of the samples. The values thus obtained were compared with an ANOVA test [29]. For further study the series of measurements made by the most experienced observer were used. The statistical package employed in the entire study was SPSS Statistics 18.0.

## Results

### Coating Characterization

**Figure 3:**
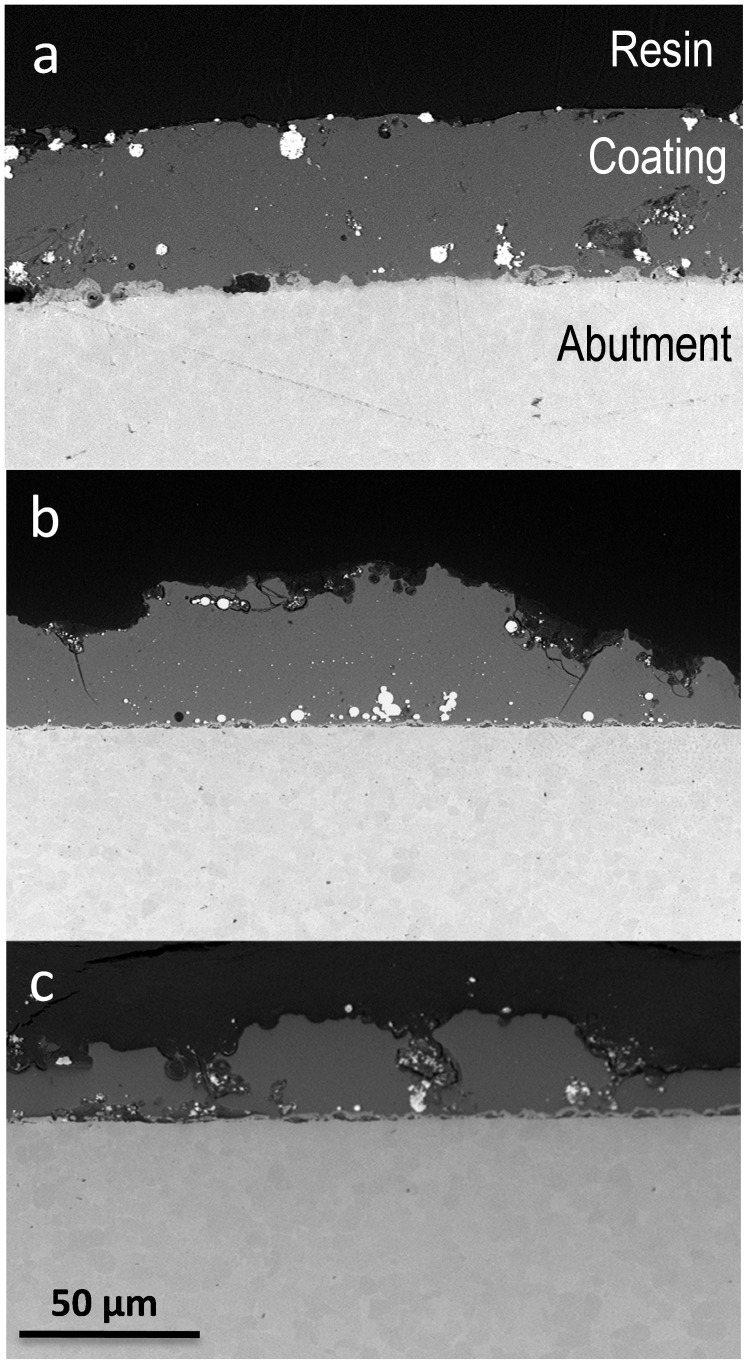
Scanning electron micrographs of cross section of the coated abutment: a) not implanted specimen, b) buccal side of the implanted specimen and c) lingual side of the implanted specimen.

A SEM image of a polished cross section of the abutment is shown in Fig. 3. During firing at 980°C the soda-lime glass containing silver nanoparticles has flown wetting the metal surface and establishing a strong joining with the abutment surface [10]. The silver particle size ranges between 20–90 nm. Very few agglomerates (0.5–8 µm) are also present. The average coating thickness corresponding to starting coating was found to be 51±14 µm. Buccal side coating and lingual side coating after euthanasia were found to be: 44±14 µm and 26±15 µm respectively. Some defects and cracks can be observed (Fig. 3). The differences were significant for a P≤0.01 (t test), therefore the null hypothesis (1) was discarded.

### Histology Description

**Figure 4:**
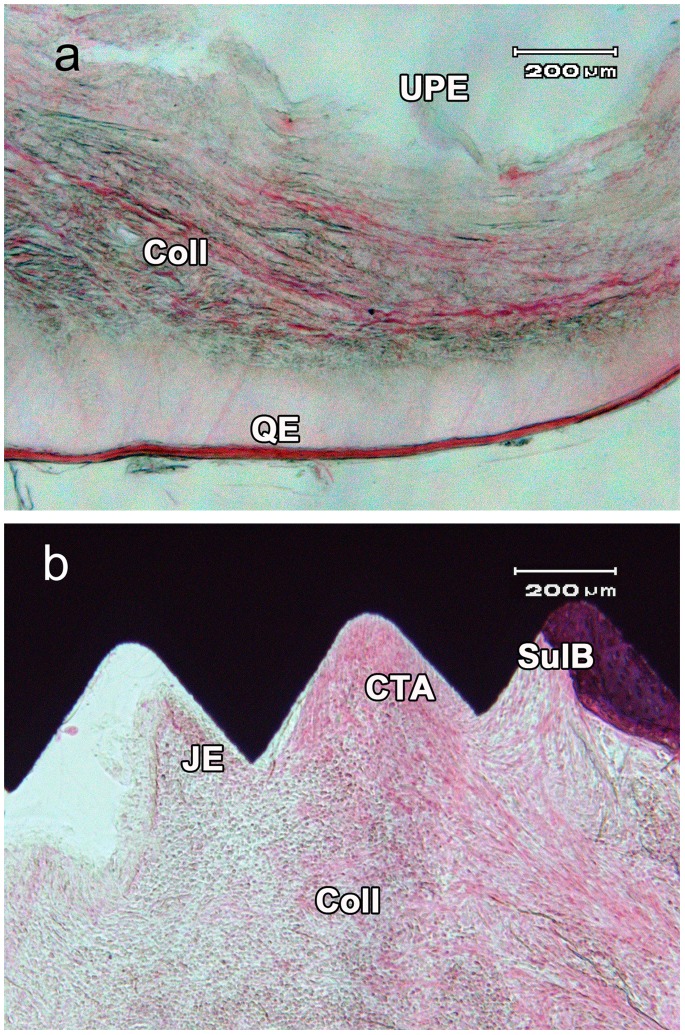
Peri-implantitis detail: a) uncoated specimen, note the ulcerated buccal pocket epithelium (UPE), the infiltration of inflammatory cells and the disruption of collagen network (Coll), and the edematized queratinized epithelium (QE). b) Coated specimen, note the connective tissue attachement (CTA) and junctional epithelium (JE), the crestal bone-implant contact (SulB), the collagen disruption and inflammatory infiltration.

A histology sections for uncoated (Group A) and coated (Group B) abutment are showing in Figure 4a and 4b respectively. In both A and B group specimens an advanced peri-implantitis was stablished. In all the studied preparations persistence of deep peri-implant pocket with its hypertrophic epithelium was observed. The pocket epithelium showed ulcerative lesions with hemorrhagic and inflammatory component and surface infiltration of polymorphonuclears (PMN) and lymphocytes. Ulcers showed a fibrinoid content with purulent accumulations of granulocytes and lymphocytes. There was an abundant proliferation of vascular buds in peripheral areas and extensive infiltration of lymphocytes and plasmocytes with a minor component of PMN leukocytes in the ulcus basal area. A hypertrophy of the peri-implant keratinized mucosa with intra-epithelial edema, submucosal vascular proliferation, disruption of the network of collagen and mononuclear cell infiltration of the submucosa layer have been also observed. Bone resorption both in buccal and lingual bone crests was observed.

The implant surface in contact with the epithelial tissue was covered with a layer of mixed bacterial plaque with a relevant component of hyphae in the proximity of the abutment. There was an accumulation of mineralized supra and sub gingival plaque in both groups. In this particular point no differences between coated and uncoated implants were observed.

There were no statistically significant differences in the frequencies of appearance of peri-implantitis categorical lesions between the two groups. The ulcerated pocket epithelium (UPE) was more frequent in the group of coated implants but without statistical signification. The infiltration of the mucosa and the breakdown of the collagen matrix were present in all studied samples (Table 2). There were a significant higher proportion of lymphocytes in the infiltrate of uncoated implants, but there were no statistical differences in the proportion of PMN and plasmocytes (Table 3).

**Table 2:** Peri-implantitis categorical scores.

	ULCUS EPIT	MUCOUS INFILT	COLAGEN DESESTRUC	INTRABON POCKET	VASCULAR PROLIFERATION
(GROUP A) UNCOAT	18	20	20	12	20
(GROUP B) COAT	30	38	38	19	38
SIGNIFχ2	0.250	CTE.	CTE.	0.328	CTE.

Note that infiltration mucosae, collagen disruption and vascular proliferation are present in all the samples in both groups. Group A: n = 20. Group B: n = 38.

**Table 3:** Counting of inflammatory cells.

	N	PMN	LIMPHOCYTES	PLASMOCYTES
(GROUP A)UNCOAT	20	34.0±15.1	21±11.6	44±8.2
(GROUP B) COAT	38	36.9±18,1	15±4.8	49±16.9
SIGNIFχ2		0.534	0.009	0.180

Celular proportions and signification. N:number of samples, PMN: proportion of polymorphonuclear.

### Bone Histometry and Statistical Analysis

**Figure 5:**
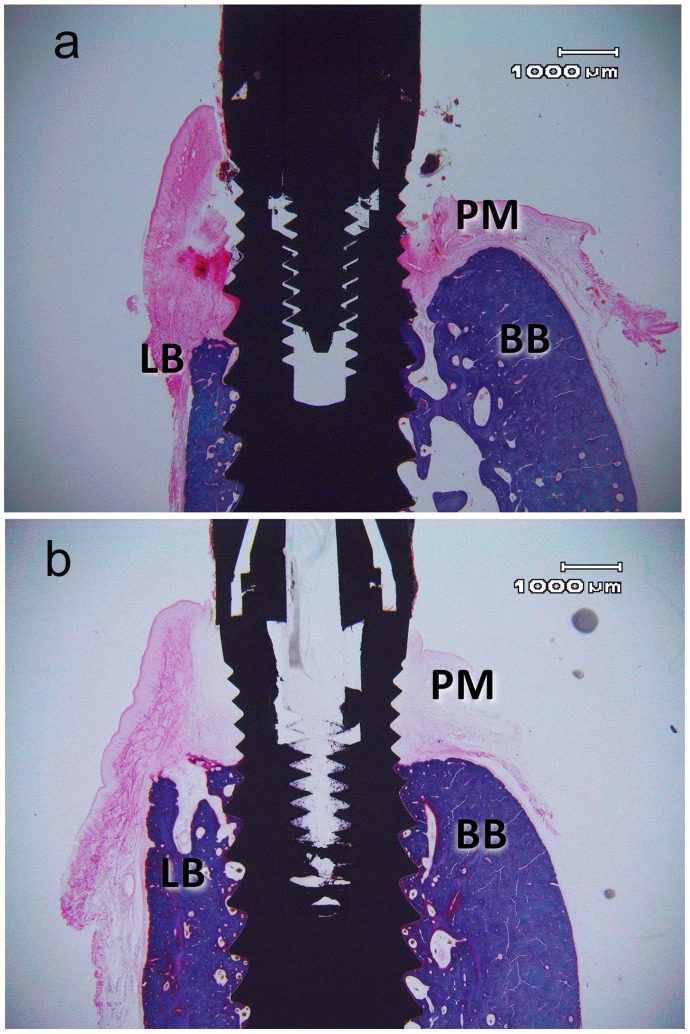
Representative buccal-lingual sections from: a) uncoated abutment (group A) and b) coated abutment (group B). Harris Haematoxylin and Weatley Trichromic stain. (PM: peri-implant mucosa; BB: buccal bone; LB lingual bone).

Representative buccal-lingual sections of uncoated abutment (group A) and coated abutment (group B) are showing in Figure 5a and 5b respectively. Bone level alterations and gingival dimensions are showing in Table 4. The interobserver measurement error (ME) obtained was 3.6%, The intraobserver error was 2.5% and 1.3%. All values exhibit a normal distribution. In the entire sample a higher bone loss on the lingual (2.9±0.63 mm) than in the buccal (2.6±0.56 mm) side with a significance level of p≤0.001 was found. Given this significance buccal and lingual data were compared independently. This asymmetric loss was less significant in the group B (coated) than in the group A (uncoated). The ANOVA analysis of mean bone recession showed a statistically significant difference in the lingual (P = 0.045) but not in the buccal area. In post hoc of the lingual area the paired t test showed that the lingual bone loss in the coated implant abutments (GROUP B1, B2) was significantly lower than in those without coating (group A) (P<0.05) (Table 4). There was no significant difference between the mean bone recession in both coated positions (groupB1, and B2 P = 0.145). The null hypothesis 2 of same bone loss in both groups was rejected. No significant differences were found in the bone recession given variable or animal, or the side of the jaw, or position in the jaw. The gingival margin (Gin) was significantly placed more occlusally in the lingual side than in the buccal side in the two types of transepithelial abutment (p<0.001). There were no significant differences in the buccal or lingual gingival margin position between coated implants and control as well as the test between each other (B1–B2). The gingival margin level is not altered by the coating (Table 4).

**Table 4:** Bone level alterations and gingival dimensions.

	N	J-Sul B (L)	J-Sul B (B)	SIGNIF	Gin-CrB (L)	Gin-CrB (B)	SIGNIF
(GROUP A)	10	3.2±0.71	2.8±0.20	0.004	3.7±0.83	1.6±2.6	<0.001
UNCOAT							
(GROUP B1)	10	2.8±0.53	2.5±0.27	0.033	3.4±0.77	1.9±0.58	<0.001
COAT							
(GROUP B2)	9	2.7±0.56	2.6±0.19	0.045	3.7±0.59	1.8±0.68	<0.001
COAT							
SIGNIF A-B1		0.045	0.098		0.087	0.101	
SIGNIF A-B2		0.039	0.125		0.101	0.144	

Bone buccal/lingual and gingival buccal/lingual means and signification. N: number of specimens, J: abutment-fixture junction, SulB: marginal position of bone-implant contact, L: lingual, B: buccal, Gin: gingival margin and CrB: crestal bone.

## Discussion

The loss of soft tissue sealing around the implant is a very important aspect in the development of peri-implantitis [14], [15]. The epithelial sealing is considered nowadays as one of the most fragile point of the integration process [12], [15]. Peri-implantitis experimental models are based on the attack against junctional epithelium and peri-implant biological width by a bacterial colonization belonging to the ligature and the subsequent deposit of supra and subgingival plaque [6], [8], [19], thus triggered a process of bone destruction which, after a certain time, progresses independently of the permanence of the ligature [6]. The use of transepithelial abutments with a biocide coating can protect the mucosa seal in a similar way to the effect of an antibiotic application depot [7], [8]. This coating can increase the surface roughness of the abutments, which according to some studies would be an aggravating factor for peri-implantitis [5], [16], even the roughness of the surface appears to be a factor that favors the periimplant lesion development [17]. The coating used in this study has a rougher surface (1±0.2 microns) than the one of polished titanium (0.5±0.3 microns) [21]. In the present study we have also stated that the aging of the coating increases the roughness of the surface, developing the presence of defects and cracks (Figure 3). It is possible that this loss of material would be in relation with the continuous lingual abrasion and the masticatory activity. This would explain the greater loss of coating on the lingual side of the abutments (approx 35% of thickness) as compared to the loss in the vestibular side (12% thickness) (Fig. 3). The repeated tongue mechanical trauma, on the damaged epithelial sealing also could explain the increased bone loss in the entire lingual area of the specimens. It could also be related to the tongue action the more occlusal position of the gingival margin at the lingual area in both groups. In the abutment coating loss may also play a summation role the chemical micro-erosion caused by peri-implantitis, as it was already described in hydroxyapatite coatings [18].

The coating of the abutments with a possible increase of the roughness of the surface versus the polished finishing does not appear to play a role for the exacerbation of peri-implantitis. In the present work, after a long-term, the coated group (B) display a lower bone loss than those connected to polished abutments. Perhaps the noxa induced by the experimental model exceeds the potential benefit of the polished and finished surface, and plaque eventually settles on all surfaces [30]. One aspect clearly stated in the present work is that despite the aggression of peri-implantitis, implants which have been connected to coated abutments have a less bone loss in the long term. It should be considered that these implants in the healing phase after connecting the abutment, when establishing the biological width [12], experience a greater bone loss [21]. That is, there is a greater apical recession of tissues at this early stage, but later, after the induction of peri-implantitis, final bone recession is less. It could be said that the first contact with the coated abutments causes a major recession but in a second stage, the presence of biocide contains the bone loss. In a previous work, the radiographic imaging morphometry showed that the biological width setting was more traumatic in implants with coated abutments [21]. Given this greater initial loss caused by the biological width setting, insertion loss attributable to peri-implantitis (after week 24) is distinctly lower in coated abutment implants.

It has been observed the formation of a more symmetrical cone resorption for implants with coated abutments. This can be due to a reduced activity of the periimplant injury. From a histopathological point of view no significant differences between both groups in establishing a peri-implantitis with all their own characteristics [4] can be mentioned. The major lymphocyte infiltration observed in the uncoated group seems to have no special relevance; there are no concurrent differences in the plasmocytes and in PMN that could indicate a lower stage in the peri-implantitis process. The increased presence of ulcerated pocket epithelium in the coated group also seems to be an isolated factor, because it is not associated to an alteration in the gingival dimensions.

Possibly the aggressiveness of the caused peri-implant lesion out-exceed the protective effect of the coating, as the mineralized plaque deposition occurs equally in both groups. The observed difference of bone loss has to be considered as an important and relevant fact taking into account that literature usually consider peri-implantitis, once established, tends to be very refractory to any local topical application to exclude the surgical approach [4], [5], [6] as well as experimental peri-implantitis models come a time, progressing independently to the permanence of the noxa [19]. In this regard the high silver nanoparticles biocidal capacity [9] could act as an element capable of constraining the hard tissue destruction.

It is important to point out that in this study randomization in the position of the implants was not performed. Although the data may be biased by this decision should be aware that given the homogeneity in size and age of the dogs, and the lack of significance in the edentulous ridge thickness, it appears that none of the three positions has been benefited from a greater amount of bone; vascularization differences appear also be influenced by lateralization patterns [24] which however in the present design are diminished. Moreover, the different possible tongue action between different positions in a small size edentulous gap seems negligible. Differences were always found between the control and coated group and never between.

One aspect to be considered is the observed loss of the abutments coating. In this sense future studies to analyze whether this loss is mainly due ether to mechanical aggression, to a chemical attack or both, and how each of which influences the loss of the layer. Also relevant is the study of possible systemic effects caused by the vehiculization and swallowing of the biocide layer by the animal, given that recently some concerning was reported about the toxicity of the continuous ingestion or penetration through mucosa or skin of the nanoparticles and its long-term accumulation in the organism [31], [32].

## Conclusions

Within the limits of this animal study, it could be concluded that implants with coated by soda lime glass containing silver nanoparticles titanium abutments are capable to constrain the bone loss experimentally induced by peri-implantitis. This particular coating not only decreases the total bone recession caused by the induced peri-implantitis but also causes a less pronounced asymmetry with a more regular bone resorption crater. It has been also proved that this coating was mechanically unstable versus the direct wear of the rugged beagle lingua and the masticatory activity, presenting aging with cracking and losing of thickness.
